# Severe Bradycardia Possibly due to a Local Anesthetic Oral Mucosal Injection during General Anesthesia

**DOI:** 10.1155/2015/896196

**Published:** 2015-02-25

**Authors:** Kenichi Satoh, Ayako Ohashi, Miho Kumagai, Hideki Hoshi, Kousei Otaka, Shigeharu Joh

**Affiliations:** ^1^Division of Dental Anesthesiology, Department of Reconstructive Oral and Maxillofacial Surgery, School of Dentistry, Iwate Medical University, Iwate 020-8505, Japan; ^2^Division of Special Care Dentistry, Department of Developmental Oral Health Science, School of Dentistry, Iwate Medical University, Iwate 020-8505, Japan; ^3^Division of Oral and Maxillofacial Surgery, Department of Reconstructive Oral and Maxillofacial Surgery, Iwate Medical University, Iwate 020-8505, Japan; ^4^Department of Anesthesiology, Omagari Kosei Medical Center, Akita 014-0027, Japan

## Abstract

Local anesthesia may induce systemic complications leading to parasympathetic activity leading to bradycardia and hypotension. We report a case of a 50-year-old man undergoing dental surgery under general anesthesia who experienced severe bradycardia and hypotension after local anesthesia infiltration. Concerns regarding the utilization of a relatively large lumen injection needle for local anesthesia during general anesthesia are discussed.

## 1. Introduction

Local anesthesia oral mucosal infiltrations have the potential to induced systemic complications [1, 2]. Such factors as vasovagal reflex and anxiety secondary to injection pain fear can trigger increased intense parasympathetic activity which may lead to bradycardia and hypotension [3]. However, to our knowledge, bradycardia induced by an intense noxious stimulus when the infiltration of local anesthetic into the oral mucosa under general anesthesia has not been previously reported. We report a case in which severe bradycardia is associated with the infiltration of local anesthetic into the oral mucosa under general anesthesia.

## 2. Case Presentation

A 50-year-old man who weighed 56.1 kg was scheduled to undergo extraction of his right mandibular wisdom tooth and excision of a tumor. He had no past medical history and had not been prescribed any drugs. His preoperative electrocardiogram was within normal limits and showed a normal sinus rhythm, a PR interval of 0.162 seconds and a QT interval of 0.387 seconds. All laboratory values were within the normal range, and the patient had no known drug allergies.

Atropine (0.2 mg) and midazolam (3.0 mg) were intravenously administered in the operating room. Anesthesia was induced with propofol (110 mg), fentanyl (50 *μ*g), and rocuronium bromide (40 mg); after endotracheal intubation, anesthesia was maintained with sevoflurane (1–1.5%) in oxygen (40%) and air (60%), fentanyl (50 *μ*g), and remifentanil (0.2 *γ*). The patient's hemodynamics were stable for the subsequent 20 minutes: heart rate (HR) was 50–55 bpm (normal sinus rhythm), systolic BP was 83–90 mm Hg, diastolic BP was 43–50 mm Hg, ETCO_2_ was 39 mmHg, and SpO_2_ was 100%. The patient's vital sign, ECG, ETCO_2_, and SpO_2_ were continuously monitored. The oral and maxillofacial surgeon infiltrated the tissue around the right aspect of the wisdom tooth and tumor with 5 mL of 1% lidocaine with 1/100,000 adrenaline (50 mg of lidocaine and 50 *μ*g of adrenaline) over a period of 20 seconds, and with a 23-gauge needle and strong pressure by surgeon's hand. Specifically the proximal side gingiva of the tooth was infiltrate into the underside of the periosteum with stronger pressure.

The patient's HR slowly decreased from 62 bpm to 36 bpm within 2 minutes. The sinus rhythm was observed but there was not bigeminy or atrial flutter on ECG. His BP remained approximately 80/43 mm Hg and his mean BP was 74 mm Hg. Atropine sulfate (0.3 mg) was administered intravenously, and his HR was restored to 67 bpm within 1 min; at this time, systolic BP was 116 mm Hg, diastolic BP was 75 mm Hg, mean BP was 89 mm Hg, and the SpO_2_ and ST-T segment were unchanged. End-tidal CO_2_ increased from 39 to 46 mm Hg. The patient's hemodynamics were stable for the remainder of the procedure: HR was 59–64 bpm, systolic BP was 82–103 mm Hg, diastolic BP was 41–58 mm Hg, and SpO_2_ was 100%. The surgery was successfully completed approximately 30 minutes after the episode of bradycardia.

## 3. Discussion

Severe bradycardia is associated with the infiltration of local anesthetic into the oral mucosa under general anesthesia. Anxiety, fear, and pain associated with clinical treatment can trigger an intense parasympathetic state leading to bradycardia and hypotension in an awake or sedated patient [3], resulting in a vasovagal reflex. In head and neck surgery, Doyle and Mark [4] reported that reflex bradycardia and sinus arrest could occur during a variety of surgical procedures, from neurosurgery to general abdominal, laparoscopic, ophthalmic, and facial surgery, in most cases, a vagally-mediated reflex was implicated. In oral and maxillofacial surgery, the trigeminocardiac reflex or oculocardiac reflex may occur in patients undergoing Le Fort *Ι* osteotomy [5], midface fracture reduction [6], elevation of zygomatic fractures [7], and temporomandibular joint insufflations [8]. This reflex is caused by traction of the extraocural muscles or compression of the eyeball during down fracture, repositioning, and manipulation, leading to a decrease in pulse rate by the efferent portion of the vagus nerve from the cardiovascular center of the medulla to the heart [9]. However, in the present case, only the oral mucosa of the mandible was infiltrated with local anesthetic, and so we do not think that the patient's response can be explained by the oculocardiac reflex.

If the patient is unconscious under general anesthesia, he should not feel pain. General anesthetics can inhibit sensory tract, cerebral cortex, and neurotransmission in spinal but there is a peripheral activation of nociceptors under general anesthesia; therefore, it is a possibility that an intense noxious stimulus indicating a peripheral activation of nociceptors associated with infiltration into oral submucosa can trigger an intense parasympathetic state leading to bradycardia.

The oral and maxillofacial surgeons typically need to infiltrate local anesthetic into the oral mucosa to decrease surgical bleeding, lessen mucosal congestion, and maintain a clear field of view. When we think about the effects of adrenaline contained lidocaine, we have usually experienced hypotension by induced adrenaline and the increase in HR. The cause of this increase in HR mainly involves a baroreceptor reflex that decreases the blood pressure and gently stimulates *β*_2_-receptors. We do not think a baroreceptor reflex occurred in this case. We confirmed that this infiltrated local anesthetic was adrenaline contained lidocaine immediately after this episode.

Regarding the possible influence of the general anesthesia pharmacotherapeutics as such atropine, midazolam, propofol, fentanyl, rocuronium bromide, sevoflurane and remifentanil, and local anesthetic lidocaine, we do not know clearly whether these drugs could be the cause or even partially influence the bradycardia. There is possibility that all these drugs have the cause or even partially influence the bradycardia but immediately after the infiltration of local anesthetic into the oral mucosa, we think that this bradycardia is induced by an intense noxious stimulus.

It is our opinion that infiltration of local anesthetic into oral mucosa with a large needle and strong pressure by surgeon's hand should be avoided. During dental treatments, the sting from the needle is occasionally associated with systemic complications, such as the vasovagal reflex, hyperventilation syndrome, ischemic heart disease, and arrhythmias [1, 2]. Satoh et al. [10] reported transient asystole at the time of needle puncture in a dental surgery patient under intravenous sedation. Suddenly, severe bradycardia can occasionally occur in an otherwise stable patient when a needle used for local anesthetic administration enters the oral submucosa during oral and maxillofacial surgery. The resulting hemodynamic effects are variable and difficult to anticipate. Generally, in awake patients the dentist very carefully infiltrates local anesthetic into oral submucosa with a small needle (28 or 30 gage needle), not strong pressure and slowly (1.8 mL per 60 seconds). But under general anesthesia surgeons usually infiltrate with a large needle (23 gauge) and strong pressure and rapidly (10 mL per within 30 seconds) because they think all sensitivity in oral mucosa is blocked by general anesthesia. It was a possibility that bradycardia in our case was induced by an intense noxious stimulus when infiltrated into the oral submucosa with large needle, strong pressure by hand and rapidly. We do not yet have enough data to judge whether the effects from needle puncture differ depending on the region of the body where the needle is introduced. Therefore, we would specifically recommendation the use a smaller gauge needle, which cannot generate as much pressure and stimulation.

In conclusion, severe bradycardia is associated with the infiltration of local anesthetic into the oral mucosa under general anesthesia and the infiltration of local anesthetic into oral mucosa with a large needle and strong pressure by surgeon's hand should be avoided. It is prudent to recognize the possibility of this complication when a needle is introduced into the oral submucosa during general anesthesia.
